# Wastewater Surveillance Detected Carbapenemase Enzymes in Clinically Relevant Gram-Negative Bacteria in Helsinki, Finland; 2011–2012

**DOI:** 10.3389/fmicb.2022.887888

**Published:** 2022-06-02

**Authors:** Ananda Tiwari, Jaana Paakkanen, Monica Österblad, Juha Kirveskari, Rene S. Hendriksen, Annamari Heikinheimo

**Affiliations:** ^1^Department of Food Hygiene and Environmental Health, Faculty of Veterinary Medicine, University of Helsinki, Helsinki, Finland; ^2^Expert Microbiology Unit, Finnish Institute for Health and Welfare, Kuopio, Finland; ^3^HUSLAB, Helsinki, Finland; ^4^Antimicrobial Resistance Unit, Finnish Institute for Health and Welfare, Turku, Finland; ^5^Technical University of Denmark, National Food Institute, WHO Collaborating Center for Antimicrobial Resistance in Foodborne Pathogens and Genomics, Kongens Lyngby, Denmark; ^6^Finnish Food Authority, Seinajöki, Finland

**Keywords:** Carbapenemase, Guiana extended-spectrum, *Klebsiella pneumoniae*, *Enterobacteriaceae*, CHROMagarKPC, Wastewater-based epidemiology

## Abstract

Antimicrobial resistance profiling of pathogens helps to identify the emergence of rare or new resistance threats and prioritize possible actions to be taken against them. The analysis of wastewater (WW) can reveal the circulation of antimicrobial-resistant bacteria (ARB) and antimicrobial resistance genes (ARG) among the catchment communities. Here, we analyzed WW influent samples to determine the prevalence of carbapenemase genes-carrying Gram-negative bacteria (Carba-GNB) in Helsinki, Finland. This study set important historical reference points from the very early stage of the carbapenemase era, during the period 2011–2012. A total of 405 bacterial isolates grown on CHROMagarKPC (*n* = 195) and CHROMagarESBL (*n* = 210) from WW influent samples were collected between October 2011 and August 2012 and were analyzed. The bacterial DNA from the isolates was extracted, and the prevalence of carbapenemases genes *bla*_KPC_, *bla*_NDM_, *bla*_GES_, *bla*_OXA-48_, *bla*_IMP_, *bla*_IMI_, and *bla*_VIM_ were screened with multiplexed PCR. All carbapenemase-positive isolates were identified taxonomically to species or genus level with matrix-assisted laser desorption ionization-time of flight mass spectrometry (MALDI-TOF MS). The nucleic acid extraction was successful for 399 isolates, of which 59 (14.8%) were found to carry carbapenemase genes. A total of 89.8% of the carbapenemase positive isolates (53 out of 59) were obtained from CHROMagarKPC plates and only 10.2% (six out of 59) were obtained from CHROMagar ESBL plates. Among the Carba-GNB isolates, 86.4% were *bla*_GES_ (51 out of 59), 10.2% were *bla*_KPC_ (six out of 59), and 3.4% were *bla*_VIM_ (two out of 59). The most common carba-gene, *bla*_GES_, was carried by 10 different bacterial species, including *Aeromonas* spp., *Enterobacter* spp., and *Kluyvera* spp.; the *bla*_KPC_ gene was carried by *Escherichia coli*, *Klebsiella pneumoniae*, and *Kluyvera cryocescens*; and the *bla*_VIM_ gene was carried by *Aeromonas hydrophila*/*caviae* and *Citrobacter amalonaticus*. This study emphasizes that wastewater surveillance (WWS) can be an additional tool for monitoring antimicrobial resistance (AMR) at the population level.

## Introduction

Antimicrobial agents are an important part of modern medicine and have saved millions of people and animals from various infections (WHO, 2018). However, extensive use of such agents has been selected for antimicrobial resistance (AMR) in many pathogens against many antimicrobial agents; so treating infections caused by such AMR pathogens has become increasingly difficult (WHO, 2018). The increase of AMR demands a wide variety of alternative antimicrobial agents, and again the excessive use of such agents further increases AMR, which threatens the entire medical system. Therefore, the World Health Organization has declared AMR a global public health threat (WHO, 2020).

Comprehensive information on AMR of different pathogens helps doctors select the right antibiotic prescription, avoid unnecessary use of ineffective antibiotics, and early preparation for treatments so that efficient treatment and recovery of individuals are more likely. Further such information helps alert authorities to the emergence of rare or new resistance threats as well as helps to prioritize actions to be taken and to evaluate the outcomes of earlier interventions. Currently, a clinical surveillance approach is used, which demands large numbers of samples from individual patients to provide epidemiologically relevant data, and which in turn requires huge amounts of resources and infrastructure (WHO, 2018; Aarestrup and Woolhouse, 2020; ECDC, 2020). Instead, monitoring municipal sewage known as wastewater surveillance (WWS) offers an economical way of observing the occurrence of antimicrobial-resistant bacteria (ARB) and antimicrobial resistance genes (ARG) in entire populations of wastewater catchment areas (Khan et al., 2018; Hendriksen et al., 2019; Hutinel et al., 2019; Aarestrup and Woolhouse, 2020; Huijbers et al., 2020; Blaak et al., 2021). It is possible because, ARB, together with human pathogens from all infected individuals (symptomatic, asymptomatic, pre-symptomatic, and post-symptomatic), is excreted through feces, urine, nasal mucus, and sputum from households, hospitals, and nursing homes, and end up in the municipal sewage system.

Further, wastewater effluents released from treatment plants are a potential environmental source of ARB and ARG (Karkman et al., 2018, 2020). From a one health perspective, such discharges of clinically relevant ARB and ARG pose a considerable health risk to both humans and animals. Therefore, knowing the prevalence of clinically relevant AMR and related genes in wastewater may also help in controlling their circulation in the environment and saving human and environmental health. The current wastewater treatment technology is not sufficient for the complete removal of AMR (Karkman et al., 2018), as the technology is specialized in the reduction of macro-nutrients such as carbon and phosphorus.

β-lactams are the most widely used antibiotic class in many countries, including Finland (WHO, 2018; ECDC, 2020). Resistance to this group of antibiotics by pathogenic bacteria poses a considerable threat to clinical patient care, public health, and animal health (Bonomo, 2017; Cherak et al., 2021). Carbapenems belong to a class of β-lactam antibiotics that present a wide spectrum of antibacterial activity, and they are a treatment of choice for serious infections caused by extended-spectrum beta-lactamase (ESBL) positive pathogens (Papp-Wallace et al., 2011; Elshamy and Aboshanab, 2020). The selective ecological pressure created by the extensive use of these groups of antibiotics accelerates the proliferation of ESBL-producing and multidrug-resistant pathogens, by suppressing antimicrobial susceptible communities and providing more ecological space for AMR communities (Villar et al., 2013; Jean et al., 2015; Codjoe and Donkor, 2017; Nasri et al., 2017; Cherak et al., 2021). Such, the emergence of ESBL-producing and multidrug-resistant pathogens may further accelerate the consumption of carbapenem antibiotics. There is a limited number of antimicrobial agents, such as polymyxins, tigecycline, fosfomycin, and aminoglycosides available for the treatment of bacteria with reduced susceptibility to carbapenems or carbapenem-resistant bacteria, and most of them are used only on serious hospital infections (Codjoe and Donkor, 2017; Sheu et al., 2019). Therefore, resistance to carbapenem signifies resistance to all available antibiotics and causes infections to become nearly untreatable (Codjoe and Donkor, 2017; Elshamy and Aboshanab, 2020).

The production of β-lactamase enzymes is one of the major mechanisms for the development of β-lactam-resistance (Elshamy and Aboshanab, 2020). Carbapenemases are among the most versatile beta-lactamases that hydrolyze most beta-lactam antibiotics and weaken the antibiotic effects against target pathogens (Codjoe and Donkor, 2017; Cherak et al., 2021). In general, clinically relevant carbapenemase groups *Klebsiella pneumoniae* carbapenemase (KPC), Guiana extended-spectrum (GES), Verona integron-encoded metallo-*β*-lactamase (VIM), New Delhi metallo-β-lactamase (NDM), imipenem-resistant *Pseudomonas* (IMP), and oxacillinase (OXA-48) are reported elsewhere (Queenan and Bush, 2007; Papp-Wallace et al., 2011; Nasri et al., 2017). This study investigated the monthly variation in the prevalence and abundance of carbapenemase-producing bacterial isolates by using CHROMagar ESBL and CHROMagar KPC plates (CHROMagar™, Paris, France) and carbapenemase genes; *bla*_KPC_, *bla*_GES_, *bla*_VIM_, *bla*_NDM_, *bla*_IMP_, and *bla*_OXA-48_ with multiplex PCR in wastewater influent of the Viikinmäki wastewater treatment plant (WWTP) in Helsinki, Finland during a pioneering study setting from the very early stage (2011–2012) of the carbapenemase era. Then, the wastewater-based results were compared with clinically reported cases during the important historical reference point.

## Materials and Methods

### Sample Collection and Analysis

**Figure 1:**
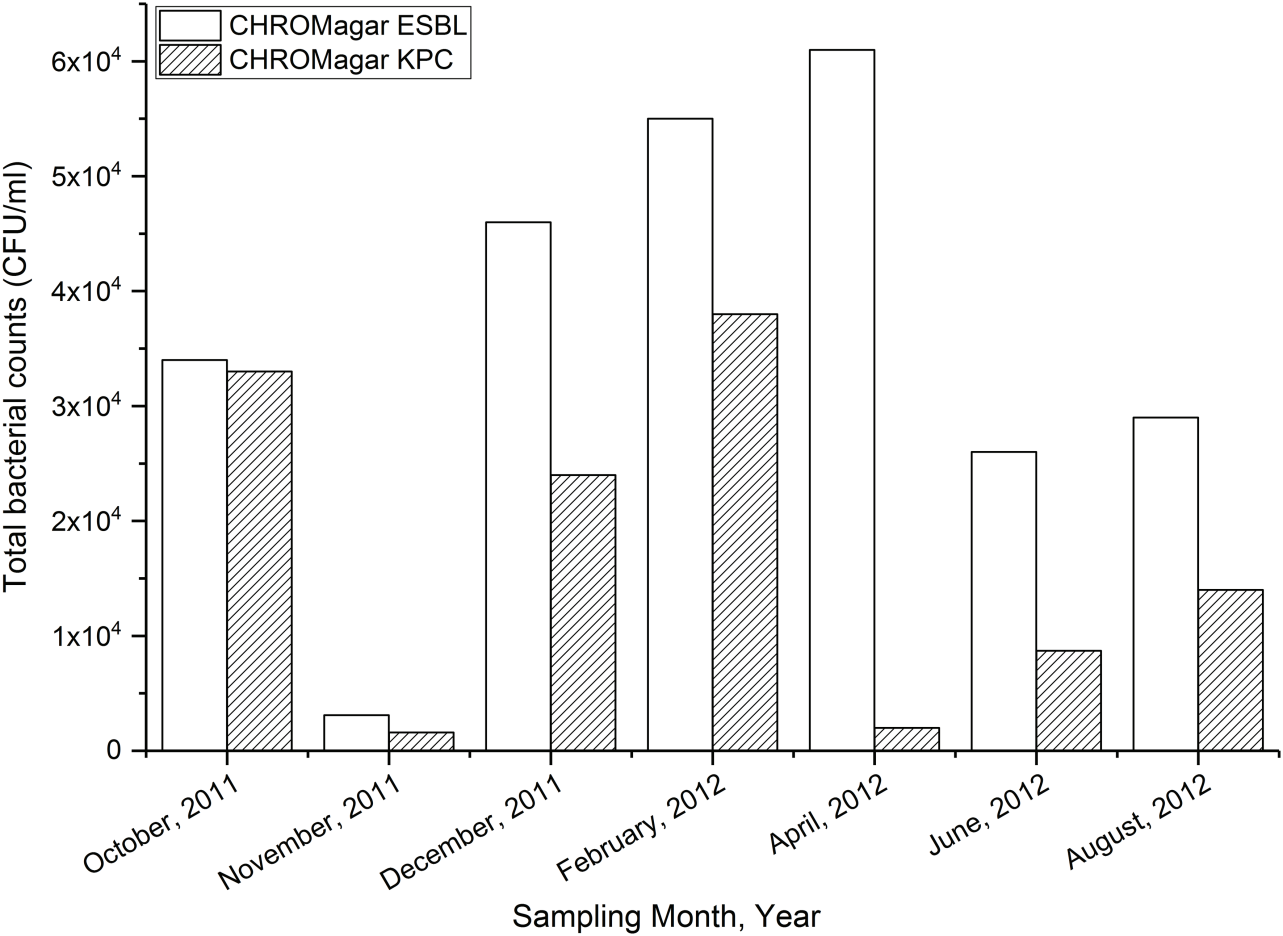
The monthly variation in the bacterial growth on CHROMagar ESBL and CHROMagar KPC media.

A total of seven 24-h composite wastewater (WW) influent samples (untreated municipal sewage) were collected in seven different months between October 2011 and August 2012 (Figure 1), from Viikinmäki WWTP in Helsinki, Finland. On average, the daily influent volume at the Vikinmäki WWTP is about 296,000 m^3^ (Tiwari et al., 2022a). The WWTP collects sewage from about 800,000 inhabitants (14.5% of the total population of Finland), including domestic households, nursing homes, primary health care centers, and hospitals in Helsinki and neighboring municipalities, namely Kerava, Tuusula, Järvenpää, Sipoo, and the eastern half of Vantaa. A 1-L sample of WW was transported in cooling boxes (~4°C) to the University of Helsinki, the department of food hygiene and environmental health, and was preferably analyzed within 6–8 h.

Initially, WW samples were serially diluted with peptone water. A 100 μl volume of each dilution concentration (original, 1/10, 1/100, and 1/1000) was inoculated on CHROMagar ESBL and CHROMagar KPC plates (CHROMagar™, Paris, France) with a spread plate technique, following the instructions provided by the manufacturers for isolating and enumerating the prevalence of ESBL-producing and carbapenem-producing bacteria, respectively. The plates were incubated at 37°C for 18–24 h, after which colonies were counted and expressed as colony-forming units (CFU) per milliliter (ml). The primary phenotypic identification of bacterial isolates was made based on the colony-color chart provided by the manufacturer (red: *Escherichia coli*; metallic blue: *Klebsiella* spp., *Enterobacter* spp. and *Citrobacter* spp.; and light-yellow/translucent cream: *Pseudomonas* spp. and *Acinetobacter* spp.). A total of 60 colonies were selected, 30 from the CHROMagar ESBL plates and 30 from the CHROMagar KPC plates, from all sampling days except only 15 colonies on October 2011 from CHROMagar KPC. An equal proportion of each colony color group (red, blue, and light yellow) was randomly picked and sub-cultured on Tryptone soya agar.

### DNA Extraction and Multiplex PCR

Subsequently, the isolates were transferred to Eppendorf tubes with 100ml of PCR water and heated to 100°C for 15 min. Cell debris was removed by centrifugation (13,000 rounds per minute, for 2 min), and the supernatant was stored at −20°C. DNA extracts from all isolates were screened to determine the presence of carbapenemase genes with QuantiTect Multiplex PCR (NoROX, QIAGEN), by using Applied Biosystem™ (ABI) BigDye Terminator v3.1 cycle sequencing kit following the procedure as explained in an earlier publication (Pasanen et al., 2014). The primers and probes of targeted carbapenemase gene families used in this study (*bla*_KPC_, *bla*_NDM-1_, *bla*_GES_, *bla*_OXA-48_, *bla*_IMP_, and *bla*_VIM_) are listed in Table 1. Negative (DNA-free water) and positive controls (PCR 1 positive control strain *bla*_KPC_ and in PCR 2 positive control strain *bla*_NDM-1_) were used during each multiplex-PCR experiment. Amplification conditions: 95°C for 10 min; 30 cycles of (95°C for 20 s, annealing temperature for 30 s, and 58°C for 30 s).

**Table 1:** Primers and probes used in multiplex PCR test.

Gene	Primer	Amplicon	Reference
*bla*_KPC_	KPC-F: TCGCTAAACTCGAACAGG KPC-R: TACTGCCCGTTGACGCCCAATCC	785	Monteiro et al., 2009
*bla*_NDM-1_	NDM-F: TTGGCCTTGCTGTCCTTG NDM-R: ACACCAGTGACAATATCACCG	82	Monteiro et al., 2012
*bla*_GES_	GES-F: CTATTACTGGCAGGGATCG GES-R: CCTCTCAATGGTGTGGGT	594	Monteiro et al., 2012
*bla*_OXA-48_	OXA-48-F: TGTTTTTGGTGGCATCGAT OXA-48-R: GTAAMRATGCTTGGTTCGC	177	Monteiro et al., 2012
*bla*_IMP_	IMP-F: GAGTGGCTTAATTCTCRATC IMP-R: AACTAYCCAATAYRTAAC	120	Mendes et al., 2007
*bla*_VIM_	VIM-F: GTTTGGTCGCATATCGCAAC VIM-R: AATGCGCAGCACCAGGATAG	382	Mendes et al., 2007

### Identification of Bacterial Isolates

All carbapenemase genes-carrying Gram-negative bacteria (Carba-GNB) identified with the multiplexed-PCR tests were streaked onto bovine blood agar plates and incubated at 37°C overnight for identification with matrix-assisted laser desorption ionization-time of flight mass spectrometry (MALDI-TOF MS) using a VITEK MS (bioMérieux). A score value of 2.0–3.0 was considered high and thus a confident match and was set as the criterion.

## Results

The temporal variations in bacterial counts on the selective plates are presented in Figure 1. Bacterial counts on both media were lowest from a sample collected in November 2011. The count was highest in CHROMagarESBL plates but still low in CHROMagarKPC plates in the sample collected in April 2012 (Figure 1).

Carbapenemase genes (*bla*_CARBA_) were detected in 14.8% of the total isolates screened (59 out of 399). A total of 89.8% of the isolates positive with *bla*_CARBA_ genes (53 out of 59 isolates) grew on CHROMagarKPC plates, and 10.2% were found on CHROMagarESBL plates (six out of 59 isolates; Table 2). In total, 27.6% of isolates on CHROMagarKPC (53 out of 192 isolates) and 2.9% of isolates on CHROMagarESBL (six out of 207 isolates) were positive for the targeted *bla*_CARBA_ genes. Among gene types, 86.4% were identified as *bla*_GES_ (51 out of 59), 10.2% were identified as *bla*_KPC_ (six out of 59), and 3.4% were identified as *bla*_VIM_ (two out of 59; Table 2). Among the six carbapenemase positive isolates obtained from CHROMagarESBL, five were *bla*_GES_ and one was *bla*_VIM_ (Table 2).

**Table 2:** Gram-negative bacterial isolates carrying various *bla*_CARBA_ genes screened from a total of 399 isolates obtained from CHROMagar extended-spectrum beta-lactamase (ESBL) and CHROMagar *Klebsiella pneumoniae* carbapenemase (KPC) media.

Species/Genus	Family	Carbapenemase genes types						
		*bla*_KPC_	*bla*_NDM-1_	*bla*_GES_	*bla*_OXA-48_	*bla*_IMP_	*bla*_IMI_	*bla*_VIM_
*Enterobacter amnigenus*	*Enterobacteriaceae*	0	0	1	0	0	0	0
*Enterobacter* spp. (3√)	*Enterobacteriaceae*	0	0	9	0	0	0	0
*Citrobacter amalonaticus* (√)	*Enterobacteriaceae*	0	0	0	0	0	0	1
*Citrobacter brakii*	*Enterobacteriaceae*	0	0	1	0	0	0	0
*Kluyvera cryocescens* (2√)	*Enterobacteriaceae*	1	0	12	0	0	0	0
*Raoultella ornithinolytica^+^*	*Enterobacteriaceae*	0	0	1	0	0	0	0
*Klebsiella pneumoniae*	*Enterobacteriaceae*	2	0	0	0	0	0	0
*E. coli*	*Enterobacteriaceae*	3	0	0	0	0	0	0
*Raoultella* spp.	*Enterobacteriaceae*	0	0	1	0	0	0	0
*Aeromonas* spp.	*Aeromonadaceae*	0	0	8	0	0	0	0
*Aeromonas/caviae*	*Aeromonadaceae*	0	0	14	0	0	0	1
*Pseudomonas putida*	*Pseudomonadaceae*	0	0	1	0	0	0	0
*Stenotrophomonas maltophilia*	*Xanthomonadaceae*	0	0	1	0	0	0	0
Unknown	-	0	0	2	0	0	0	0
	Total isolates	6	0	51	0	0	0	2

*A mark “√” denotes isolates grown on CHROMagar ESBL; otherwise, all isolates were grown on CHROMagar KPC media*.

**Figure 2:**
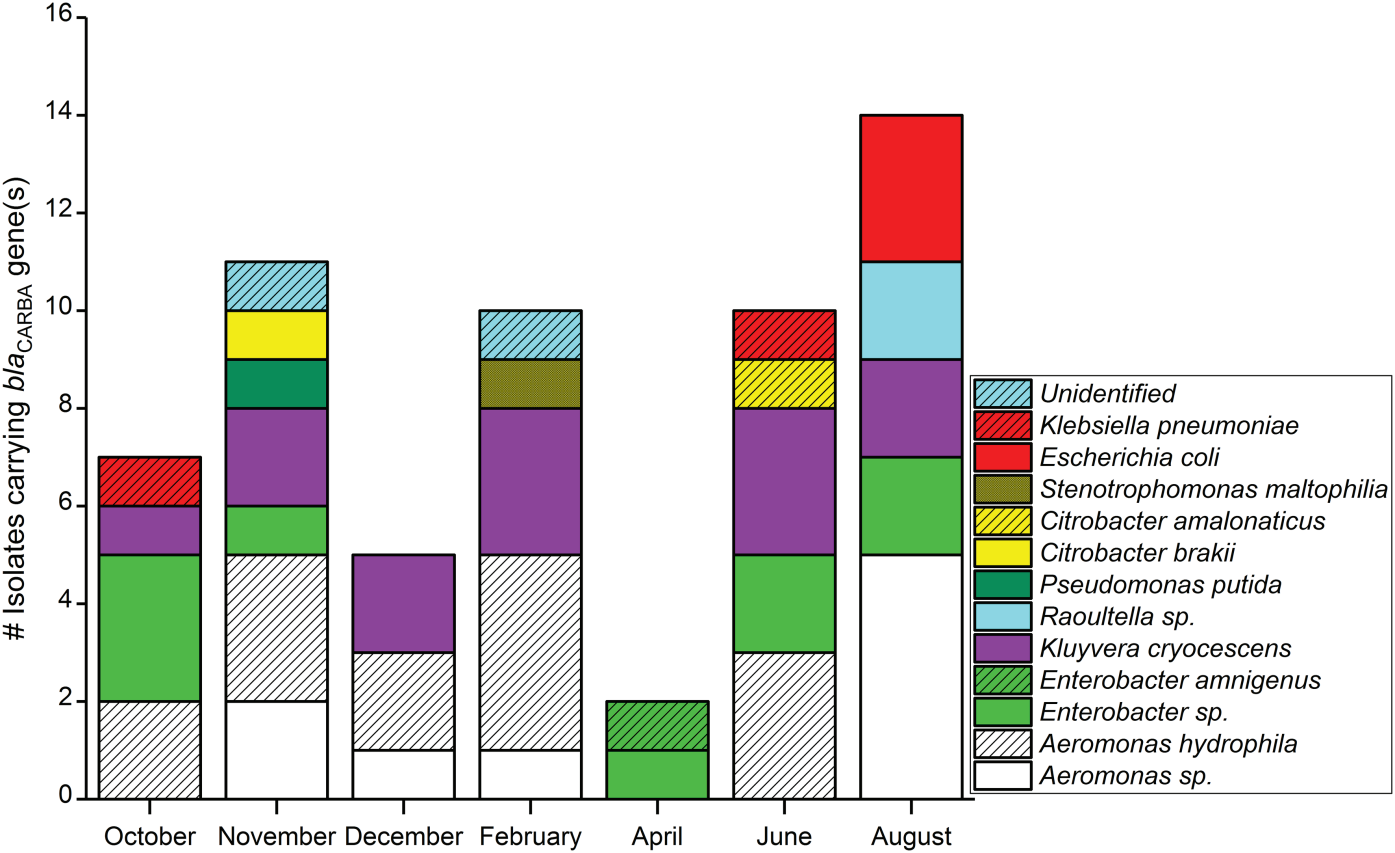
*bla*_CARBA_ gene-carrying Gram-negative bacterial isolates (*n* = 59) isolated in wastewater influent in Helsinki, Finland (2012).

Comparing monthly variations in *bla*_CARBA_-carrying bacteria, the highest number of isolates was detected in August (*n* = 14), followed by November (*n* = 11), and the lowest numbers were detected in April (*n* = 2; Figure 2). When considering only *bla*_GES_ genes, samples collected during November and August had an equal number of *bla*_GES_ positive isolates, (*n* = 13). The *bla*_KPC_ was found only in bacteria isolated from summer–spring samples (three isolates in August, two isolates in June, and one isolate in October). The two *bla*_VIM_ gene-positive isolates were collected from the June sample. Bacterial isolates carrying *bla*_NDM-1_, *bla*_OXA-48_, and *bla*_IMI_ genes were not detected over the entire study period.

### Bacterial Species and *bla*_CARBA_ Families

**Figure 3:**
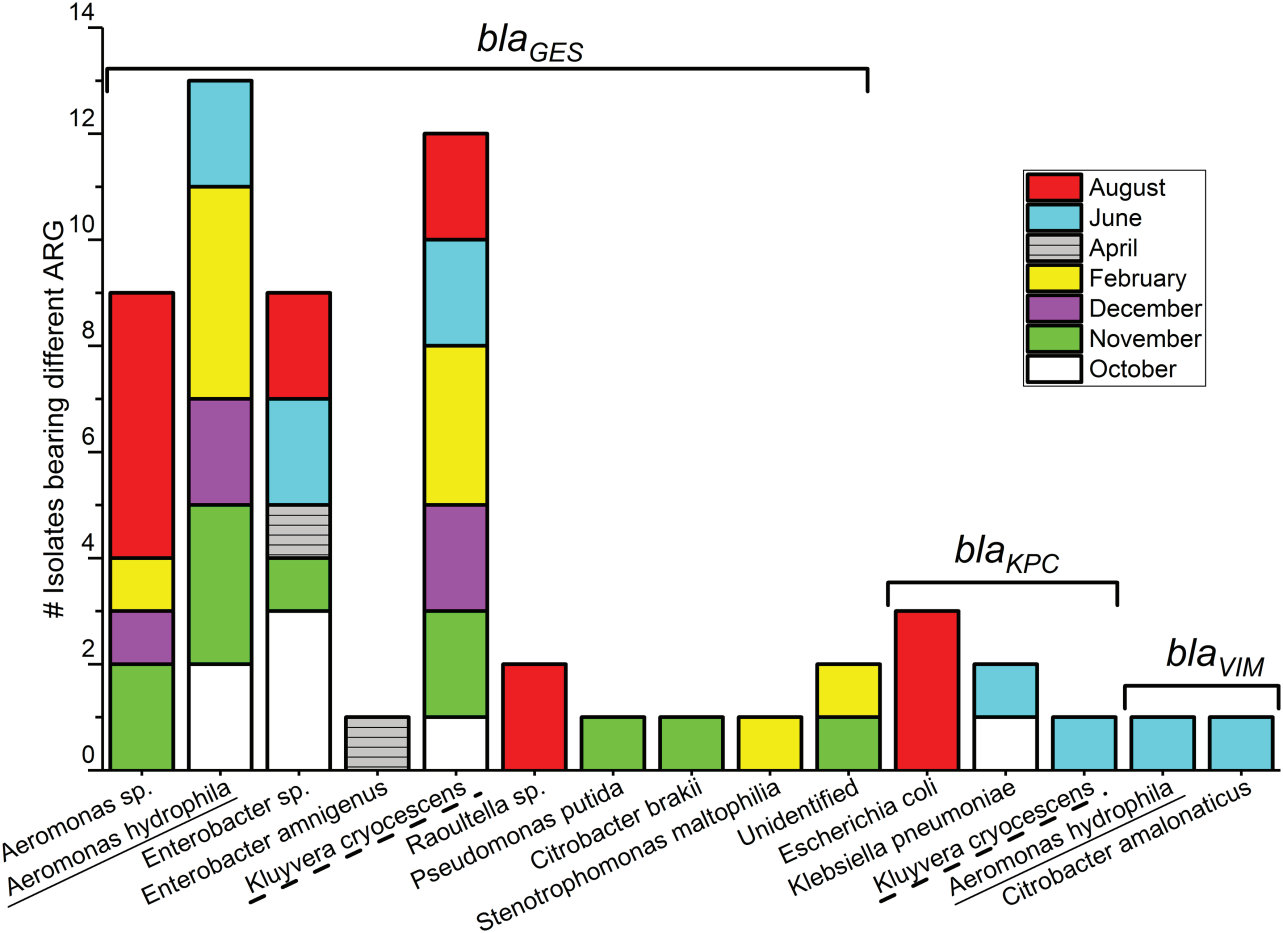
Gram-negative bacterial species carrying *bla*_CARBA_ genes, isolated from wastewater.

Among 59 Carba-GNB isolates, 39 isolates were identified to species level, 18 isolates were successfully identified only up to genus level, and two isolates remained unidentified by MALDI-TOF MS (Table 2; Figures 2, 3). Out of a total of 51 bacterial isolates carrying *bla_GES_* genes, 31 isolates were identified up to species level, 18 were identified up to genus level, and two isolates remained unidentified. Among the *bla_GES_* gene-carrying isolates identified up to species level, *Aeromonas hydrophila*/*caviae* and *Kluyvera cryocescens* were the most common, with 14 and 12 isolates respectively, and among isolates identified only up to genus level, *Enterobacter* spp. and *Aeromonas* spp. were the most common, with nine and eight isolates, respectively. The isolate identification method used in this study (MALDI-TOF MS) was not able to differentiate between *A. hydrophila* and *A. caviae* isolates. There was one isolate of *Citrobacter brakii*, *Enterobacter amnigenus*, *Pseudomonas putida*, *Raoultella ornithinolytica*, *Stenotrophomons maltophilia*, and *Raoultella* spp. in each group. The *bla*_KPC_ was the second most prevalent gene type detected in this study with a total of six isolates, three isolates were identified as *E. coli*, two isolates as *Klebsiella pneumoniae*, and one isolate as *Kluyvera cryocescens*. The two *bla*_VIM_ isolates were identified as *Aeromonas hydrophila*/*caviae* and *Citrobacter amalonaticus*.

## Discussion

Antibiotic-resistant Gram-negative bacteria carrying *bla_GES_* carbapenemase genes were dominant, and *bla*_KPC_ and *bla*_VIM_ were also recorded in some isolates from wastewater influent samples collected between October 2011 and August 2012 from the Viikinmäki WWTP Helsinki, in the capital region of Finland while using CHROMagar ESBL and CHROMagar KPC plates (CHROMagar™, Paris, France). To our knowledge, these are the earliest carbapenemase genes detected in non-clinical samples in Finland. In agreement with our result, a study conducted in 2020 also reported *bla*_GES_ and *bla*_VIM_ as the most abundant *bla*_CARBA_ genes in hospital wastewater in Helsinki (Majlander et al., 2021). In contrast with our methodology, they extracted environmental DNA directly from wastewater filtrate and screened for various *bla*_CARBA_ genes with a high-throughput qPCR method. Another study reported a wide variety of ARG in the WW influent of Viikinmäki WWTP using direct extraction of environmental DNA from WW, and gene detection using qPCR (Karkman et al., 2016). A study from the Netherlands, based on direct extraction of environmental DNA, using high-throughput qPCR, reported *bla*_GES_ from both hospital and municipal sewage, but the abundance in hospital sewage was higher than in the municipal sewage (Buelow et al., 2018).

As municipal sewage is considered to provide a glimpse of the ARB and ARGs circulating in a community (Kwak et al., 2015; Hendriksen et al., 2019; Pärnänen et al., 2019; Blaak et al., 2021), our findings indicate that *bla*_GES_, *bla*_KPC_, and *bla*_VIM_ were the most prevalent *bla*_CARBA_ genes in Helsinki during the study period. Diverging from our wastewater-based finding, Österblad et al. (2012) reported a total of 17 clinically relevant *bla*_CARBA_ genes belonging to *bla*_NDM_, *bla*_OXA-48_, *bla*_OXA-181_, *bla*_VIM_, and *bla*_NMC-A_ in Finland in 2011–2012. The *bla*_GES_ types of *bla*_CARBA_ have rarely been reported in clinical cases in Finland (Supplementary Figure S1). Apart from clinical sources, there can be many other possible sources of *bla*_GES_ detected in wastewater in our study. It could be contributed from environmental sources and human normal flora that are not monitored *via* clinical isolates. Further, one systematic review reported *bla*_GES_ genes to be reported less often *bla*_CARBA_ genes in many other geographical areas (Halat and Moubareck, 2020). Another study reported *bla*_GES_ carrying *Enterobacteriacease* isolates from non-clinical sources in hospital wastewater (White et al., 2016). Another study suspected the ubiquity of bacterial communities carrying *bla*_GES_ genes in different environments (Manageiro et al., 2014). A total of 23 *bla*_GES_ sub-types have been identified to date and most of them are not clinically monitored, as they are weak carbapenem hydrolyzers and some of them are extended-spectrum beta-lactamase producers but do not produce carbapenemase (ECDC, 2013; Nordmann and Poirel, 2014; Naas et al., 2016).

About 70% of the individuals infected with Carba-GNB in 2012 were travelers returning from foreign countries, mostly from Asia or southern Europe (Räisänen et al., 2020). Among the most frequent clinical *bla*_CARBA_ gene types (Supplementary Figure S1), *bla*_KPC_ is endemic in the United States, *bla*_NDM_ is endemic in South Asia, and *bla_OXA-48_* is endemic in South Asia and northern Africa (Nordmann and Poirel, 2014). Clinical cases of *bla_NDM_* type of infection increased rapidly after the year 2011 and *bla*_KPC_ types of infection after 2015 (Supplementary Figure S1). The first clinical isolates of *bla*_KPC_ in Finland were reported in 2009 and were reported each year between 2012 and 2018 (Räisänen et al., 2020). The clinically reported *bla*_CARBA_ genes *bla*_NDM_, *bla*_OXA-48_, *bla*_OXA-181_, *bla*_VIM_, and *bla*_NMC-A_ during the year 2011–2012 were not detected in wastewater.

We isolated *bla*_CARBA_ genes from *Aeromonas hydrophila*/*caviae*, *Aeromonas* spp., *Enterobacter amnigenus, Enterobacter* spp., *Kluyvera cryocrescens, Raoultella* spp., *Pseudomonas putida*, *Citrobacter brakii*, and *Stenotrophomons maltophilia*. Many of these bacteria were reported in earlier studies as *bla*_CARBA_ carriers (Nordmann and Poirel, 2014; Codjoe and Donkor, 2017; Xu et al., 2018; Cherak et al., 2021). However, the choice of identification method or selective isolation media might affect the taxonomic results of a study. ESBL media are sensitive for picking up any common carbapenem resistance (not IMI/NMC-A without concomitant ESBL), even often low-level OXA-48/181, but due to lack of specificity to carbapenemases, it is not sensitive for selecting heteroresistance when carbapenemase producers are not abundant in the presence of ESBL species harboring same color (Hornsey et al., 2013). In one recent study, Majlander et al. (2021) confirmed *Acinetobacter baumannii*, *K. pneumoniae*, and *Pseudomonas aeruginosa* from the hospital wastewater of Helsinki as Carba-GNB with a qPCR method. They confirmed the prevalence of these bacteria by gene profiling, and they did not report the prevalence of the bacterial groups detected in our study. Many studies have reported *E. coli*, *K. pneumoniae*, *P. aeruginosa*, and *A. baumannii* in WW samples carrying major clinically relevant *bla*_CARBA_ groups (Nordmann and Poirel, 2014; Naas et al., 2016; Codjoe and Donkor, 2017; Blaak et al., 2021). Xu et al. (2018) reported *bla*_KPC-2_ genes from *C. freundii* and *Aeromonas* spp. isolates from river sediment.

The most common clinically reported Carba-GNB isolates in Finland in 2011–2012 were *K. pneumoniae*, *E. coli*, and *C. freundii* from 16 clinically ill patients (Supplementary Figure S2). However, based on a random test in clinically suspected cases, out of *E. coli* (*n* = 3,161), *K. pneumoniae* (*n* = 536), and *P. aeruginosa* (*n* = 327) clinical isolates screened with a susceptibility test, ~0, ~0 and 6.1% were resistant to carbapenems, respectively, in 2011–2012 (ECDC, 2013). Finland had a lower percentage of carbapenem-resistant clinical isolates than many other European Union (EU) member countries. For example, in 2012, the EU average was <0.1% among *E. coli*, 6.2% among *K. pneumoniae*, and 17.1% among *P. aeruginosa* isolates resistant to carbapenems (ECDC, 2013). The highest percentage, 67% of tested *K. pneumoniae* isolates, was reported from Greece (ECDC, 2013).

The World Health Organization has classified carbapenem-resistant *Enterobacteriaceae* (CRE) as a critical priority, driving the need to develop new antibiotics (Davies and Simeon, 2017). Some bacterial genera confirmed as Carba-GNB in our study, such as *Kluyvera* spp. and *Aeromonas* spp., have been described in the environment and reported to be potential opportunistic pathogens (Parker and Shaw, 2011; Davin-Regli et al., 2019; Li et al., 2019). Perhaps, some *bla*_CARBA_ genes are related to aquatic environments and only occasionally cause infection. Many of these bacterial species could be asymptomatically carried by humans in their intestines, and thus be found in wastewater. Clinical isolates can differ from wastewater-based isolates. The clinical isolates are pathogenic, but wastewater-based isolates better represent symbiotic and normal human bacteria.

The idea of wastewater-based epidemiology has been effective for monitoring SARS-CoV-2 (Hokajärvi et al., 2021; Tiwari et al., 2022a), as viruses are highly host-specific and do not multiply outside a host. But in the case of AMR, the interpretation is not so straightforward. For example, the frequently detected genes in our study, *bla*_GES_, *bla*_KPC_, and *bla*_VIM_, are plasmid-encoded carbapenemases (Naas et al., 2016; Halat and Moubareck, 2020), and these are highly prone to horizontal gene transfer (HGT). Current knowledge about the frequency of HGT in wastewater is limited (Fahrenfeld and Bisceglia, 2016). Bacteria forming biofilms and multiplying on surfaces in distribution pipes may affect the occurrence of bacteria in the wastewater (Singh et al., 2017). Nutrient-rich wastewater containing varying levels of biocidal chemicals might assert varying selection pressure on its bacterial content (Fahrenfeld and Bisceglia, 2016). Additionally, the possible contribution of carbapenemase genes from zoonotic sources related to domestic pets (dogs and cats), human food, commercial farm animals, and runoff from agricultural areas cannot be ruled out (Grönthal et al., 2018; Kurittu et al., 2021a,b). However, our study site was an urban area without significant commercial farms, so there might be no significant contribution from commercial farms. Current WWS of AMR cannot distinguish between the potential sources of the detected ARB and ARG. Based on a one-health perspective, such collected ARB and their associated genes in WWTP can reach the public through food and drinking water if sufficient precautions are not taken (Kurittu et al., 2021a; Tiwari et al., 2022b). Effective wastewater treatment can reduce such risks (Karkman et al., 2016; Barancheshme and Munir, 2018).

The low detection of *bla*_CARBA_ isolates in November samples was unknown. The temporal variation of *bla*_CARBA_ isolates in different months cannot be confirmed due to having only one sample on each month. However, earlier studies reported, that the possible higher abundance of multi-drug resistance and ESBL bacteria in winter and spring months than in summer months can be due to higher antibiotics consumption (Miller et al., 2014; Schages et al., 2020). Further, the HGT rate can be higher during cold than in warm temperatures (Miller et al., 2014). Detecting only 2.9% Carba-GNB on CHROMagar ESBL was not surprising, as this medium is specialized for the selective isolation of ESBL/AmpC bacterial groups. The CHROMagar KPC is specific but insensitive to low-level carbapenem resistance as it has an elevated ertapenem concentration (Hornsey et al., 2013). At the time of this study, more sensitive CHROMagar mSuperCARBA was not available. Kwak et al. (2015) reported that 34 and 55% of total *E. coli* isolates grown on CHROMagar from municipal sewage influent and hospital wastewater, respectively, were positive for ESBL genes with a PCR method (Kwak et al., 2015). A limitation of our study is that only genes targeted by the primers used could be found.

Two *bla*_CARBA_ isolates could not be identified even to the genus level, possibly because the MALDI-TOF MS technology used in this study was developed for identifying clinical isolates, and thus had a lower detection capacity for environmental isolates.

## Conclusion

This study identified common Carba-GNB and associated genes in WW influent in the Helsinki area, a pioneering study setting important historical reference points from the very early stage (2011–2012) of the carbapenemase era. The most dominant gene was *bla*_GES_, detected mainly in *Aeromonas hydrophila*/*caviae*, *K. cryocescens*, and *Enterobacter* spp. The findings of this study, together with the clinical reports from the following years indicate a steady increase in Carba-GNB infections in Finland and support WWS as a potential preparedness tool for AMR surveillance. WWS could supplement the clinical surveillance approach in monitoring the possible circulation of AMR in communities and shows potential as a public health tool. Future studies could cover larger geographical areas of Finland to obtain a more representative national picture. The detection of wide varieties of ARG in sewage influent samples underlines the importance of proper sewage treatment to avoid the dissemination of ARG in the environment and reduce public health risks.

## Data Availability Statement

The original contributions presented in the study are included in the article/**Supplementary Material**; further inquiries can be directed to the corresponding authors.

## Author Contributions

AH, MÖ, RH, and JK conceptualized, acquired funding, supervised the project, and allocated resources. AH and JP analyzed samples in the laboratory. AH and AT analyzed and visualized data. AT, AH, and JP drafted the initial version of the manuscript. All authors contributed to the article and approved the submitted version.

## Funding

This work was supported by the Academy of Finland postdoc funding, grant no. 261329 for conducting research and WastPan project, grant no. 339417 for manuscript writing.

## Conflict of Interest

The authors declare that the research was conducted in the absence of any commercial or financial relationships that could be construed as a potential conflict of interest.

## Publisher’s Note

All claims expressed in this article are solely those of the authors and do not necessarily represent those of their affiliated organizations, or those of the publisher, the editors and the reviewers. Any product that may be evaluated in this article, or claim that may be made by its manufacturer, is not guaranteed or endorsed by the publisher.
