# Supplementary material for: Wastewater Surveillance Detected Carbapenemase Enzymes in Clinically Relevant Gram-Negative Bacteria in Helsinki, Finland; 2011–2012
Source: Front Microbiol. 2022 Jun 2;13:887888. doi: 10.3389/fmicb.2022.887888 (PMC9201422; doi:10.3389/fmicb.2022.887888)
Supplement: Supplementary file 1 [file Table_1.DOCX]

Supplementary Material

**Wastewater surveillance detected carbapenemase enzymes in clinically relevant Gram-negative bacteria in Helsinki Finland; 2011-2012**

Ananda Tiwari^1,#^, Jaana Paakkanen^2,*^, Monica Österblad^3,**^, Juha Kirveskari^2,***^, Rene S. Hendriksen^4^, Annamari Heikinheimo^1^

^1^Department of Food Hygiene and Environmental Health, Faculty of Veterinary Medicine, University of Helsinki, Finland.

^2^ HUSLAB, Helsinki, Finland.

^3^Antimicrobial Resistance Unit, Finnish Institute for Health and Welfare, Turku.

^4^Technical University of Denmark, National Food Institute, WHO Collaborating Centre for Antimicrobial Resistance in Foodborne Pathogens and Genomics, Kongens Lyngby, Denmark.

**Current addresses:**

*Labquality Oy, Helsinki, Finland; **Environmental Agency, City of Raseborg, Raseborgsvägen 37, 10 650 Ekenäs; ***Hologic, Espoo, Finland.

**# Correspondence:**Ananda Tiwari, [ananda.tiwari@helsinki.fi](mailto:ananda.tiwari@helsinki.fi); phone: +358 442535468





**Supplementary Figure 1**. Annually reported clinical bacterial isolates carrying blaCARBA genes in Finland during 2008-2018 (Österblad et al., 2012; Räisänen et al., 2020).





**Supplementary Figure 2**. Annually reported clinical isolates of Enterobacteriaceae carrying blaCARBA genes in Finland during 2008-2018 (Österblad et al., 2012; Räisänen et al., 2020).
